# Pressure Relief, Visco-Elastic Foam with Inflated Air? A Pilot Study in a Dutch Nursing Home

**DOI:** 10.3390/healthcare3010078

**Published:** 2015-02-12

**Authors:** Martin Van Leen, Jos Schols

**Affiliations:** 1 Avoord Zorg en Wonen, Etten-Leur, 4872 CW, The Netherlands; 2 Department of Family Medicine, Faculty of Health, Medicine and Life Sciences, Maastricht University, Maastricht, 6200 MD, The Netherlands; E-Mail: jos.schols@maastrichtuniversity.nl

**Keywords:** pressure ulcers, repositioning, inflated air overlay, 3-step prevention protocol

## Abstract

Objective: There is still little evidence regarding the type of mattress that is the best for preventing pressure ulcers (PUs). In a Dutch nursing home, a new type of overlay mattress (air inflated visco-elastic foam) was tested to analyze the opportunity for replacement of the normally used static air overlay mattress in its three-step PU prevention protocol In this small pilot the outcome measures were: healing of a category one pressure ulcer, new development or deterioration of a category one PU and need for repositioning. Methods: We included 20 nursing home residents with a new category one pressure ulcer, existing for no longer than 48 h following a consecutive sampling technic. All residents were staying for more than 30 days in the nursing home and were lying on a visco-elastic foam mattress without repositioning (step one of the 3-step protocol) at the start of the pilot study. They had not suffered from a PU in the month before. The intervention involved use of an air inflated foam overlay instead of a static air overlay (normally step 2 of the 3-step protocol). At the start; the following data were registered: age; gender; main diagnosis and presence of incontinence. Thereafter; all participating residents were checked weekly for PU healing tendency; deterioration of PUs; new PUs and need of repositioning. Only when residents showed still a category one PU after 48 h or deterioration of an existing pressure ulcer or if there was development of a new pressure ulcer, repositioning was put into practice (step 3 of the PU protocol). All residents participated during 8 weeks. Results: Seven residents developed a new pressure ulcer category one and still had a category one pressure ulcer at the end of the study period. One resident developed a pressure ulcer category 2. Fifteen residents needed repositioning from one week after start of the study until the end of the study. Conclusions: Overall 40% of the residents developed a pressure ulcer. Seventy five percent of the residents started with repositioning because there was no healing tendency of their category one PU diagnosed at the start of the pilot. Because this new type of overlay mattress resulted in an increased PU incidence, and almost standard need of repositioning with accompanied high costs, this type of overlay mattress gives no benefit above the traditional visco-elastic foam mattresses in combination with the originally used static air overlay.

## 1. Introduction

In nursing home residents prevention of pressure ulcers (PUs) is very important; not only due to their frailty, but also because PUs considerably reduce quality of life and also are a very expensive problem [1,2].

Good PU preventive care may result in patient and health economic benefits. Still around 6% of the residents in Dutch nursing homes suffer from a PU (data LPZ measurements 2013) [3]. That is why adequate preventive measures remain of great importance.

Pressure relieving systems are commonly used for prevention and in the treatment of pressure ulcers. To reduce the risk of pressure ulcers, clinicians and nurses can use several types of support surfaces to redistribute pressure over a larger surface area of the patient’s body. Despite their widespread use in daily practice, there is still little scientific evidence supporting the use of these systems, except expert opinion [4,5].

In the nursing homes of Avoord Zorg en Wonen in Etten-Leur/Zundert, the Netherlands, all residents basically lie on a visco-elastic foam mattress by protocol. This strategy is based on the advices of the Dutch guideline for prevention and treatment of PUs from 2002 [6] and fits in the current (inter)national guidelines [4,7]. These guidelines also specify that repositioning always should be considered, even when residents are lying on anti PU-mattresses.

However, as shown in an article of van Leen *et al.* (2014), repositioning as a standard procedure in residents at risk of PUs is only applied in around 16% in daily practice [8]. Reasons for this low percentage are not willing to disturb the privacy and sleep of the residents and the high, physical workload of the nursing staff.

Based on two randomized controlled studies [8,9] that we performed in the Netherlands, we developed in 2004 a 3-step PU prevention protocol for the Avoord nursing homes.

These 3 steps are:

Step 1: All residents receive a standard visco-elastic foam mattress, without repositioning.

Step 2: Residents who develop signs of a possible category one PU (nonblanchable redness for a period of at least two hours), receive a static air overlay (Repose^®^, Frontier Therapeutics, Blackwood, UK) in addition to the visco-elastic foam mattress; again without repositioning.

Step 3: Residents who still develop a PU after applying step 2, will be repositioned every three hours by day and four hours during the night within 24 h.

This 3-step protocol for prevention of PUs has been structurally executed during the last 9 years [10]. The protocol has led to a structurally low nosocomial PU prevalence (between 0.1% and 2.6%), yearly measured in the first week of April.

However, in daily practice, the use of a static air overlay mattress causes some practical problems because this overlay is rather vulnerable. The mattress is made of poly-urethane and one of the disadvantages is the progressive permeability of air during life time. To reduce failure of the preventive effects of the overlay by deflation, nursing staff needs to check the inflation level every day and mostly reflation by pump is necessary (time spare 5–10 min). That is why we always have been interested in new types of air mattress that do not have this disadvantage. One of these involves the SimCair self-inflating overlay (Enmed Technolocy, Cork, Ireland at start of study, now Hill-Rom ltd., Batesville, IN, USA), a totally new concept, involving air inflated honey grade visco-elastic foam (with a honey grade look including visco‑elastic septums to store the air). The twist action high flow valves at each corner of the overlay automatically self-inflate the mattress without the need for manual or powered pumps.

In this pilot study we explored the effect of this new type of overlay mattress used as a replacement of our static air overlay mattress (used in step 2 of our protocol).

The aims of this pilot study were:

- To evaluate the clinical efficacy of a combination of a standard 15 cm visco-elastic foam mattress with an integrated static air-foam overlay mattress (SimCair^®^, Hill Rom, Batisville, IN, USA in preventing pressure ulcers
- To evaluate the need of repositioning on this type of mattress.

## 2. Subjects and Methods

### 2.1. Setting and Sample

This small observational pilot study was carried out in a nursing home of Avoord Zorg en Wonen in Zundert, the Netherlands. 20 residents, with a Braden score < 19, who developed a pressure ulcer category 1 on a visco-elastic foam mattress during the study period (September 2009–September 2012) were included, following a consecutive sampling strategy.

The inclusion criteria for residents were: age > 65, new acute pressure ulcer category 1, and informed consent of the residents themselves or in case of cognitive disorders by their representatives. The only exclusion criterion was a life expectancy of less than 3 months.

### 2.2. Intervention

Instead of the static air overlay mattress as a step 2 measure, residents received the integrated static air-foam overlay mattress.

### 2.3. Data Collection

At baseline we measured age, sex, category of pressure ulcer, presence of incontinence and the main diagnosis. From day one, PU healing tendency or deterioration and development of new PUs was assessed by trained nurses, according to the EPUAP-NPUAP PU classification [4]. A PU cat 1 was diagnosed when during two or more hours nonblanchable redness of the skin was seen.

Residents on the test mattress who developed a new pressure ulcer or showed no healing or deterioration of the included ulcer received repositioning within 24 h, as a normal prevention activity in the 3-step PU prevention ulcer protocol.

## 3. Results

Twenty residents were included and 19 finished the study period. One patient died 2 days before the end of his study period. At that moment there were no signs of a pressure ulcer. The cause of acute dying was a possible heart attack, not related to this study. The baseline characteristics of the residents are shown in Table 1. The results of the pilot study are shown in Table 2.

**Table 1:** Demographics of participating patients.

Demographics	Results
Age in years	83.4 (71–95)
Gender (females)	18
Gender (males)	2
Dementia	19
CVA	1
Category 1 PU at start	16
Category 2 PU at start	4
Category 1 PU at the end	7
Category 2 PU at the end	1 (different spot)
PU development in spite of repositioning	8
Incontinence	14
Repositioning at start study	0
Need of repositioning at the end study	15
Malnutrition state *	8

* measured with or BMI < 20 and/or weight loss more than 5% in one month/10% in 6 months.

**Table 2:** Results pilot study.

Development of PU and repositioning	Results
Healed Category 1PU without repositioning	5 (25%)
Healed Category 1PU with repositioning	15 (75%)
Category 1 PU at the end	7 (35%)
Category 2 PU at the end	1 (different spot)
PU development in spite of repositioning	8 (40%)
Repositioning at start of study	0
Need of repositioning at the end of study	19 (95%)

15 residents needed a step-3 procedure to realize PU healing seven residents developed a new pressure ulcer category one and had still a category one pressure ulcer at the end of the study period. One patient developed a new pressure ulcer category 2 in the last week of the pilot.

## 4. Discussion

The main aim of this pilot study was to evaluate the clinical efficacy of an integrated combination of a standard 15 cm visco-elastic foam mattress with an air inflated visco-elastic overlay mattress as a step 2 measure instead of the normally used combination of a visco-elastic foam mattress and a static air overlay [10]. The results show that the air inflated visco-elastic overlay mattress caused an increased percentage of category one pressure ulcers (35%) in a short time. Moreover a considerable increase of repositioning (75%) was necessary to heel the category 1 PUs visible at the moment of inclusion and during the rest of the testing period. These results are very negative compared to the usual effects of our normal step-2 strategy [10].

Because the new step 2 measure involved the use of the new mattress without repositioning, we were able to analyze the effects of this new air inflated visco-elastic overlay mattress.

In 2 RCTs with a static air overlay without application of repositioning that we performed earlier, we saw development of pressure ulcers only in 5%–7% of the residents during a period of six months [8,9]. In our 3-step PU prevention protocol, start of repositioning is the last step.

Based on a longitudinal study 2002–2011, that we performed, we concluded that start of repositioning was only necessary in 5%–10% of the participating residents. However, in this pilot study 75% of the participating residents needed additional repositioning within a week; and overall 90% needed repositioning! Repositioning often is a very uncomfortable activity and must be done by 2 nurses. Therefore in this pilot study the accompanied costs were estimated to be very high with ultimately a result that was worse.

Of course this pilot study is only a small exploratory study and therefore it is not possible to make a final hard conclusion. Yet, the results have withheld us to perform subsequently a larger longitudinal study (RCT) with this air inflated visco-elastic overlay mattress.

## 5. Conclusions

From this study, it may be concluded that the tested integrated static air-foam overlay mattress has not presented itself as a beter solution than the originally used static air overlay mattress.
